# High-Transparency and Colorless Polyimide Film Prepared by Inhibiting the Formation of Chromophores

**DOI:** 10.3390/polym14194242

**Published:** 2022-10-10

**Authors:** Chuanxiang Su, Pengjia Liu, Jingyu Yue, Hengjian Huan, Zhenghui Yang, Kai Yang, Haiquan Guo, Jianying Zhao

**Affiliations:** 1 School of Chemistry and Chemical Engineering, Shandong University of Technology, Zibo 255049, China; 2 Australian Institute for Bioengineering and Nanotechnology, The University of Queensland, Brisbane, QLD 4072, Australia; 3 State Key Laboratory of Polymer Physics and Chemistry, Changchun Institute of Applied Chemistry, Chinese Academy of Sciences, Changchun 130022, China

**Keywords:** colorless polyimides (CPIs), charge transfer complex (CTC), chromophore, end-capping

## Abstract

Colorless polyimides (CPIs) with outstanding mechanical properties are essential materials in the production of flexible display panels, foldable windows, and even spacecraft cockpits. This paper specifically elaborates that the Morkit unit, and azo and nitro chromophores are important factors contributing to yellow PI, together with the well-known charge transfer complex (CTC) theory. Three diamine monomers, two anhydrides monomers, and three blockers were used to inhibit chromophores formation and, thus, obtain CPI films. The cut-off wavelength was blue-shifts to 334 nm and the transmittance is improved to 98.9% in the UV–vis range. Mechanical and thermal properties of the CPI films are not reduced through coupling effects of the blockers. Therefore, the inhibition method of the Morkit units and chromophore groups is a promising process for preparing CPIs to be used as flexible display materials.

## 1. Introduction

In recent times, colorless polyimides (CPIs) have been widely studied for applications in prospective flexible substrates of electronic and micro-optical devices [1,2,3]. They can be used in fields where glass is usually used such as in displays [4,5], memory [6], lighting, solar cells [7], etc. CPI combines excellent thermal and chemical resistances and the outstanding mechanical and electrical properties of traditional aromatic polyimide films (PIs), as well as having the advantages of common polymer optical films [8,9]. However, the traditional PI (Kapton, poly (4,4′-oxydiphenylene-pyromellitimide) has a relatively long cut–off wavelength (~454 nm) and the film appears tan or brown, thus, affecting its optical transmittance.

In general, the yellowish PIs can be explained using charge transfer complex (CTC) theory [10,11,12], caused by the π electron transition within the conjugated polyimide molecule structure. In previous studies, researchers attempted to enhance the optical transparency of CPI by the incorporation of various moieties, such as halogen [13,14,15], a kinked monomer [16,17], hyperbranched structures [18,19], aliphatic/alicyclic segments [20,21,22], large lateral bases [23,24], fluoro-containing groups (–CF_3_) [25,26], and so forth. These molecular substituents or structures prohibit the formation of CTC induced by the electron-donating and electron-withdrawing moieties in CPIs [27]. However, aliphatic or alicyclic introduction is often accompanied by low mechanical toughness of the PI film. Another way to obtain transparent PIs is to manufacture composite PIs with inorganic materials. Hybrid components include organo-clay [28], metal and metallic oxides [29,30], boron nitride [31], carbon nanomaterials [32,33,34], nucleating agents [35], and organosilicates [36]. However, it remains a great challenge to manage the trade-off between high solubility/optical properties and other desirable properties.

**Scheme 1:**
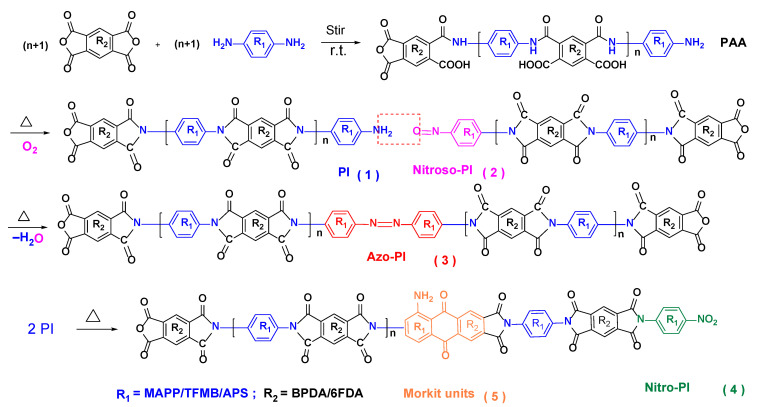
The possible colored reactions during thermal imidization of PAA to PI (**1**) with chromophores of –N=O (**2**), –N=N– (**3**), –NO_2_ (**4**), and colored Morkit units (**5**) production.

We initially aimed to synthesize CPIs by using 2,2-Bis [4-(4-aminophenoxy) phenyl]propane (BAPP) diamine and 3,3′,4,4′-Biphenyl tetracarboxylic dianhydride (BPDA), since the conjugated polyimide molecule structure is interrupted by –C (CH_3_) _2_–in BAPP segments according to the CTC theory. Yet, the obtained PIBB (polycondensation from BAPP/BPDA) films are still yellow, suggesting that other factors may also contribute to amber-colored PIs. As elaborated in Scheme 1, we hypothesize that the residual terminal amino groups (1) of the PAA can be oxidized by the trace amounts of air during the heating process or after exposure to light. The formed –N=O (2) and –NO_2_ (4) groups are two well-known chromophores that absorb in the region of 435–480 nm to remove blue light from the visible spectrum; the reflected light is recognized as being yellow. Additionally, the residual terminal amino groups (1) can react with the –N=O groups (2) to produce colored azo chromophores (3) by electrophilic addition followed by water elimination. The residual terminal anhydride groups can react with the diamine segments to produce a colored Morkit unit (5) by electrophilic substitution during the heating process. Many polymers are colored because they contain chromophores. A chromophore is a chemical group that absorbs light at a specific wavelength and imparts color to an organic molecule. Although the amount of the chromophores may not be much, the UV–vis cut-off wavelength (**λ_cut_**) and transmittance can be greatly affected by the high ε_max_ according to the Beer–Lambert law A = ε_max_ × bc. To keep the PI films from participating in the above chromogenic reactions, it makes sense to inhibit chromophores by capping the residual terminal amino and anhydride groups of the PAA. 

In previous studies, norbornene [37] or dianhydride [38] end-cappers were introduced into photosensitive polyimide’s precursor, to obtain polyimide films with desirable mechanical properties, or used in thermosetting polyimides known as bisnadimide (or PMR) resin; bisphthalonitrile [39], phenylethynyl groups [40], and acetylene-terminated polyimides [41] were also used to prepare the thermo-cured resin; polycyclic arenes were used as blockers to synthesize hyperbranched polyimides [42]; and acetic anhydride, hexahydrophthalic anhydride, o-phthalic anhydride, and endic anhydrides were used as end-capping reagents to prepare transparent PI films [43], but these anhydrides might produce the colored Morkit units as shown in Scheme 1.

In this study, we first examined a two-step capping method to improve optical properties of PI films by terminating the amino with dianhydrides and terminating the dianhydrides with glycol. Next, we utilized two diamine monomers with the electron-withdrawing effect of –SO_2_– in APS or the steric hindrance of –CF_3_ in TFMB to prevent the formation of Morkit units by inhibiting the electrophilic substitution of benzene rings in diamine when attacked by the anhydride groups. The optical absorption edge of the PI films shifts towards shorter wavelengths and the transparency of capped PI films in the UV–vis region significantly improved by the above strategies.

## 2. Experimental Section

### 2.1. Materials and Methods

2,2-Bis [4-(4-aminophenoxy) phenyl]propane (BAPP, AR); 3,3′-Sulfonyldianiline (APS); 2,2′-bis (trifluoromethyl) benzidine (TFMB); 3,3′,4,4′-Biphenyl tetracarboxylic dianhydride (BPDA, AR) and 4,4′-(Hexafluoroisopropylidene) diphthalic anhydride (6FDA, AR) were provided by Chinatech (Tianjin, China) Chemical CO. LTD. All diamines were purified by recrystallization from ethyl acetate followed by sublimation under reduced pressure. All dianhydrides were purified by sublimation under reduced pressure before use. Dimethylacetamide (DMAc) and glycol (EG, AR) were purchased from Sinopharm Chemical Reagent Co. LTD (Shanghai, China). DMAc was distilled over CaH_2_ under reduced pressure.

### 2.2. Measurements

Fourier transform infrared (FT-IR) spectra data was obtained on a Bruker vector-2 spectrophotometer (BrukerOptik GmbH, Ettlingen, Germany) (PerkinElmer Co., Ltd., Waltham, MA, USA) with a scanning range of 4000–500 cm^−1^. The optical transmittance spectra of synthesized PI films formed on silica substrates (with dimensions of 0.15 × 25 × 50 mm^3^) were measured using a UV-vis spectrophotometer (UV-3600, Shimadzu Corporation, Kyoto, Japan) in the range of 200–800 nm. The intrinsic viscosities (η_inh_) of the polyimide solutions were measured at 20 °C in a solution of DMAc (C_0_ = 0.75 g/dL) using a Ubbelohde viscometer (Julabo Inc. Seelbach, Germany). Thermogravimetric analysis (TGA, SDT650, TA Instruments, New Castle, DE, USA)was performed using an integrated thermal analyzer in the temperature range of 25–800 °C with a heating rate of 10 °C/min. The Differential scanning calorimetry (DSC) traces were obtained from a TA-Q2000 analyzer (TA Instruments, New Castle, USA) within a temperature range of 30–400 °C and STA449 (Netzsch, -Gerätebau GmbH, Selb, Germany) instruments in N_2_ with a heating rate of 10 °C/min within a temperature range of 30–800 °C, the gas flowrate was 20 mL/min. Mechanical properties of the composite films were measured using a universal testing machine (Instron-1121, Instron, Norwood, MA, USA)) at a crosshead speed of 5 mm/min under room temperature. Reported values are the average of five independent measurements. Static contact angle was measured with water by a SL200B (USA KINO, Boston, MA, USA) device; three measurements for each sample were obtained at room temperature.

### 2.3. Preparation of Polyimide Film and End-Capped Polyimide Film

**Scheme 2:**
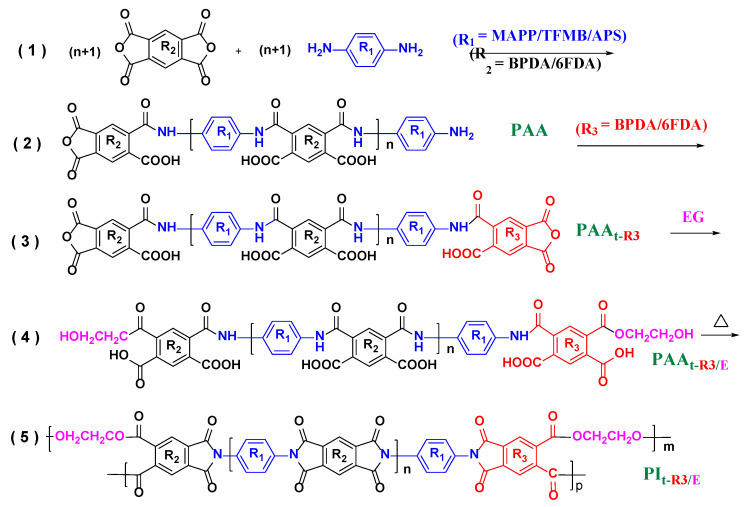
Monomer structures and highly transparent polyimide synthesis by two-step poly (amic acid) (PAA) precursor end-capping with dianhydride monomer (R3) and ethylene glycol (EG).

The synthetic two-step capping method for forming poly (amic acid) (PAA) precursors and PI films is shown in Scheme 2. The PAA was synthesized from the diamine monomer R_1_ and the dianhydride monomer R_2_. The residual amino groups in PAA can be capped by dianhydride R_3_ to produce PAA_t-R3,_ while the residual anhydride groups of PAA_t-R3_ can be blocked by EG to produce end-capped PAA _t-R3/E_. The PI_t-R3/E_ films can be made by thermal imidization of the precursor poly (amic acids) of PAA_t-R3/E_.

The synthetic method was applicable for other PIs (s stands for serial). Here we describe the poly (amic acid) synthesis of PABB_R_, PABB_t-F_, PABB_t-F/E_ (PA is the abbreviated name for poly (amic acid), BB stands for BAPP and BPDA monomers, _R_ stands for reference, t- stands for terminal, F stands for the blockers of 6FDA, and E stands for the blockers of EG) and the corresponding PI films as examples. The residual amino groups in PABB_R_ can be capped by 6FDA to produce PABBt-F and the residual anhydride groups of PABB_t-F_ can be blocked by EG to produce end-capped PABB_t-F/E_. PIBB_t-F/E_ films can then be made by thermal imidization of the precursor poly (amic acids) of PABB_t-F/E_.

Taking the synthesis of PIBB as an example, firstly, PABB_R_ was prepared under nitrogen. A total of 4.1052 g BAPP (10 mmol) was added to a three-necked flask containing 23.27 g DMAc. The mixture was stirred constantly at room temperature(r.t.) until it completely dissolved. Next, 2.9422 g BPDA (10 mmol) in 16.67 g DMAc was added into the BAPP mixture in two batches. The mixture was continuously stirred under nitrogen until it completely dissolved. Subsequently, the mixture was vigorously stirred under atmospheric nitrogen at room temperature for 24 h to obtain the PABB precursor solution.

Next, 0.0889 g 6FDA (0.2 mmol) was added into the PABB_R_ solution. The mixture was continuously stirred for 8 h to synthesize 6FDA-blocked PABB (PABB_t-F_). Then, 0.01231 g EG (0.2 mmol) was added into the PABB_t-F_ solution and continuously stirred for another 8 h to prepare the EG-capped PABB_t-F_. This was marked as the PABB_t-F/E_ precursor solution.

At last, homogeneous PABB_R_, PABB_t-F_, and PABB_t-F/E_ precursor solutions were coated on precleaned silica substrates. They were cured in a 70 °C oven for 4 h, then stepwise thermally annealed from 100 to 250 °C with an increase of 30 °C every 60 min until imidization was complete. The silica substrates were immersed in hot water at 60° C for 2 h to peel the film, and the PIBB_R_, PIBB_t-F_, and PIBB_t-F/E_ films were finally obtained.

PABB_R_ solution capped with BPDA and EG was prepared by the same process mentioned above. The only difference was that the blocker of 6FDA (0.0889 g, 0.2 mmol) was substituted by 0.0588 g BPDA (0.2 mmol). The PIBB_R_ film capped with BPDA and EG were also prepared in the same way as described earlier, and marked as PIBB_t-B_ and PIBB_t-B/E_. They were named PIBBs (PIBB serial films).

The PITF (PITF_R/_PITF_t-F_/PITF_t-F/E_) and PISF (PISF_R/_PISF_t-F_/PISF_t-F/E_) serial films were prepared using TFMB and APS as diamine monomers, respectively, 6FDA as dianhydride monomers, and 6FDA and EG as blockers. The PASF_R_/PASF_t-F_/PASF_t-F/E_ and PATF_R_/PATF_t-F_/PATF_t-F/E_ were the PAA precursor solutions of PISFs and PITFs, respectively (T, F, and S stand for TFMB, 6FDA, and APS monomers, respectively).

The PASF_R_/PASF_t-F_/PASF_t-F/E_ and PATF_R_/PATF_t-F_/PATF_t-F/E_ can be prepared with the same process mentioned earlier except by replacing BAPP (10 mmol) with APS (2.4830 g, 10 mmol) or TFMB (3.2023 g, 10 mmol). BPDA (10 mmol) was replaced by 6FDA (4.4424 g, 10 mmol) and the two step blockers were 6FDA (0.0889 g, 0.2 mmol) and EG (0.0123 g, 0.2 mmol).

## 3. Characterization Results and Discussion

### 3.1. IR Characterization of Polyimide Film

**Figure 1:**
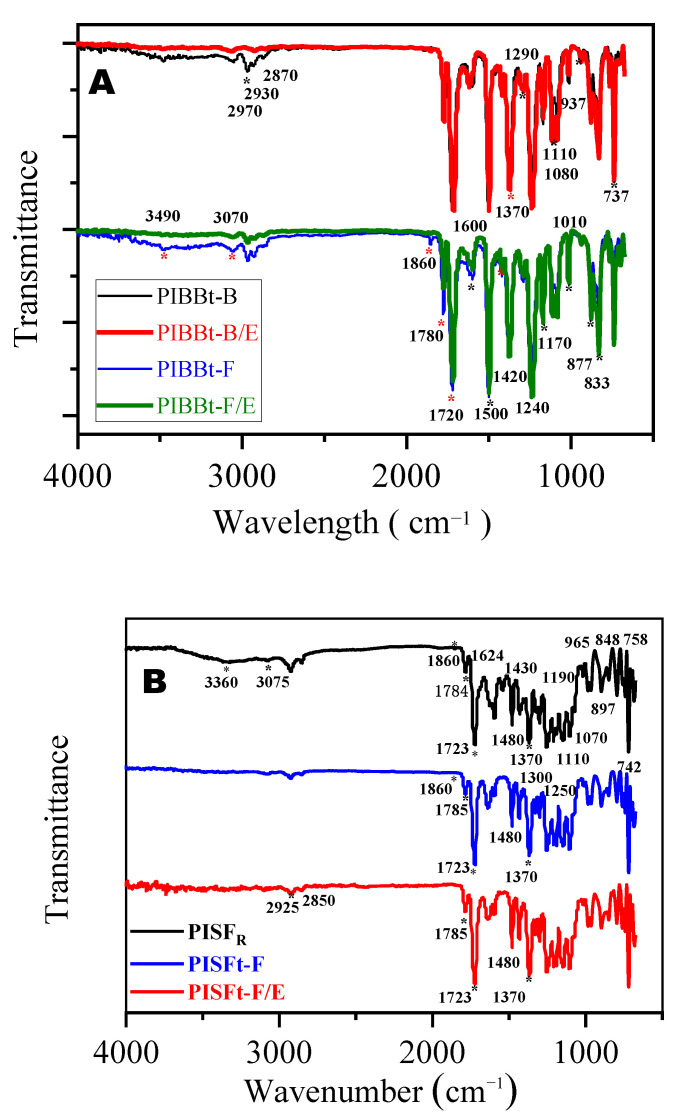
FT-IR spectra of PIBBs (**A**) and PISFs (**B**).

The molecular structures of PIBBs and PISFs were confirmed by FT-IR, as shown in Figure 1A,B. There is no absorption at 3270 cm^−1^ and 1680 cm^−1^ due to the N–H and C=O stretching vibration of the –CONH– groups in PAA. The peaks at 1720/1723 cm^−1^ and 1780/1785 cm^−1^ are due to the symmetric and asymmetric stretches of C=O, while the peaks at 1420/1430 cm^−1^ and 1370 cm^−1^ correspond to the asymmetric and symmetric stretches of C–N in the imide ring. They are observed in the PIBBs and PISFs, thus, suggesting the imidization is complete in PIs. In the IR spectra of PIBB_t-B_, PIBB_t-F_, PISF_R,_ and PISF_t-F_, there is a very weak and broad peak at 3490 cm^−1^ assigned to the hydrogen bonded O–H and N–H stretching vibration. All peaks at 1860 cm^−1^ are due to the C=O stretching vibration of the anhydride groups fading away in the PIBB_t-B/E_, PIBB_t-F/E_ and PISF_t-F/E_ after –NH_2,_ –COOH and anhydride tail groups are capped by blockers. The absorption bands at 1600 cm^−1^ and 1500 cm^−1^ are attributed to the C=C stretching vibration and bending vibration in the benzene ring of the PIs. Characteristic peaks at 3070 cm^−1^, 877 cm^−1^, 831 cm^−1^, 769 cm^−1,^ and 721 cm^−1^ are the stretching and bending vibration of theC_ph_–H in the benzene ring. The absorption bands at 1240/1250 cm^−1^ and 1110 cm^−1^ demonstrate the asymmetric and symmetric stretching vibration of C_ph_–O. The peaks at 1290/1300 cm^−1^ and 1170/1190 cm^−1^ are attributed to the asymmetric and symmetric stretching vibration of the C_ph_–N. in Figure 1A. Peaks appearing at 2970 cm^−1^ and 2870 cm^−1^ are attributed to the absorption of symmetric and asymmetric stretching vibrations of C–H in the –CH_3_ group of BAPP. The peak at 937 cm^−1^ is attributed to the bending vibrations of the O–H in the H-bonds of the two –COOH groups. In Figure 1B, the characteristic peaks at 1210 cm^−1^ are generated by C–F asymmetric stretching vibrations in the –CF_3_ group of 6FDA. The new absorption bands appearing at 987 cm^−1^ and 796 cm^−1^ are attributed to S=O and C–S stretching vibrations in –SO_2_– groups of APS segments.

### 3.2. Performance of Colorless Polyimide Film

#### 3.2.1. Optical Properties of Colorless Polyimide Films

**Figure 2:**
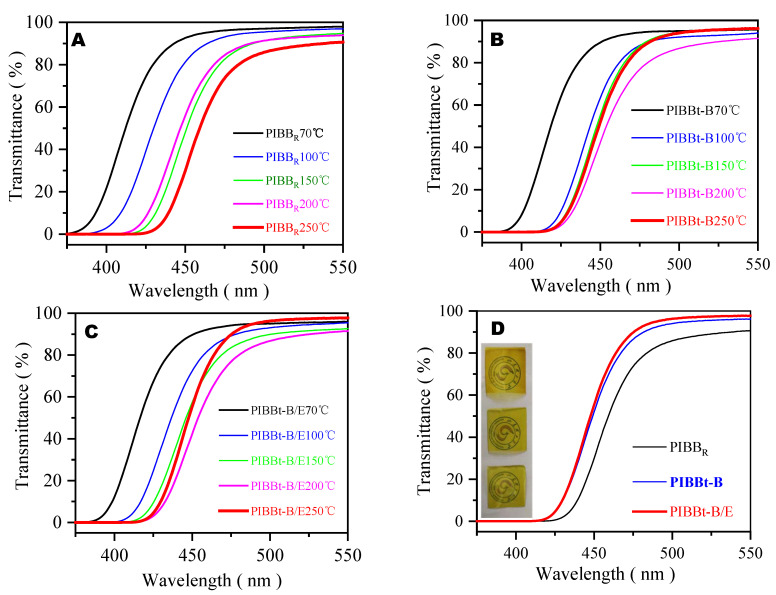
UV-vis spectra of PIBB_R_ (**A**), PIBB_t-B_ (**B**), PIBB_t-B/E_ (**C**) films treated at different temperature; UV-vis spectra comparison of PIBB_R_/PIBB_t-B_/PIBB_t-B/E_ after 250 °C treatment (**D**).

**Figure 3:**
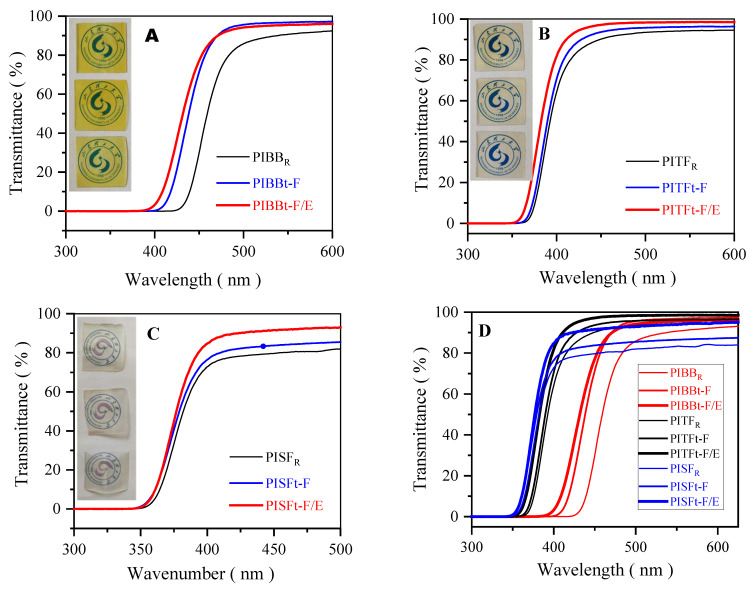
Digital photographs and UV-vis spectra comparison of the PIBBs (**A**), PITFs (**B**), PISFs (**C**) and UV-vis spectra comparison of PIBBs/PITFs/PISFs (**D**) after 250 °C treatment.

High optical transmittance is a crucial requirement for substrates and display devices. To investigate the optical performance of PI films, transmittance tests were carried out with a film thickness around 0.15 mm using ultraviolet-visible (UV-vis) spectroscopy. The important parameters to characterize the transparency of the PI films are the UV-vis cut-off wavelength (λ_cut_), the transmission at 450 nm (T_450_), and the maximum transmittance of the ultraviolet–visible band (T_max_). The test results are shown in Table 1 and Table 2, and Figure 2 and Figure 3.

The ultraviolet spectra of PI films corresponding to different heat treatment temperatures were used to verify the electrophilic substitution predicted in Scheme 1. The homogeneous PABB_R,_ PABB_t-B_, and PABB_t-B/E_ precursor solutions were coated with the pre-cleaned silica substrates. They were cured in a drying oven at 70 °C for 4 h, then thermally annealed from 100 to 250 °C with a stepwise increase of 50 °C every 60 min before finally cooling to room temperature. The UV-vis spectra of the PIBB films are shown in Figure 2, and the optical performances are listed in Table 1.

Generally, the ultraviolet cut-off wavelength (**λ_cut_**) represents the starting point of the upwards rise on the film transmittance test curve. It can be seen from Figure 2A that the **λ_cut_** of the PABB_R_ film is 362 nm after drying at 70 °C for 4 h. The film is colorless and transparent, indicating that the CTC effect of PABB_R_ is not enough to make the film yellow. However, after the PABB films were separately heated at 100 and 150 °C for 1 h, the **λ_cut_** gradually red-shifts to 370 nm and 398 nm, hence, suggesting nitro compounds or Morkit units form at 100–150 °C, turning the PABB_R_ film yellow. However, when the imide reaction of PABB films is complete at 200–250 °C to obtain the PIBB_R_ film, the **λ_cut_** of the PIBB_R_ film does not change much. This indicates that the CTC effect of the imide ring in PIBB_R_ does not make the film more yellow. Therefore, the CTC effect caused by the main chain structure of PABB or PIBB may not be the only cause of rendering colored PIBB film. Two side reactions of PABB or PIBB shown in Scheme 1 are important reasons for the yellowing of the PIBB film, and can occur during synthesis of PABB or heating of PIBB. Therefore, the two side reactions should be avoided during the preparation of CPI films.

In this research, the PABB_R_ precursor solution was subsequently capped with BPDA and EG to obtain PIBB_t-B_ and PIBB_t-B/E_ films. The UV-vis spectra and optical performances are shown in Figure 2B,C and Table 1. In comparison to PIBB_R_ films, PIBB_t-B/E_ and PIBB_t-B_ films show similar change patterns, but shorter **λ_cut_**. The T_450_ and T_max_ values both increase. There are three factors that contribute to the improved optical properties. Firstly, decreased nitro-compound content due to terminal amino groups being capped by anhydride groups of BPDA. Secondly, decreased Morkit unit formation after BPDA capped the residual terminal amino. When strong electron-donating groups (–NH_2_) are changed to the weak electron-donating amide groups (–NHCO–) or imide groups, the electron cloud density of tail diamine segments are reduced. thus. electrophilic substitution by the anhydride groups becomes difficult. Thirdly, decreased Morkit units after EG capped the anhydride groups, as residual terminal anhydride groups can react with the benzene rings in diamine segments via electrophilic substitution as predicted in Scheme 1. On the other hand, Rayleigh scattering due to ordered structures at the nanometer scale is also lowered by EG, which further improves the transparency of PIBB_t-B/E_ films. Using the two-step capping method with residual amino groups and anhydride of PAA can prevent PI films from yellowing later during the heating process. The above results demonstrate that both residual amino and dihydrates groups have significant effects on the optical properties of PI films, as well as CTC effects.

Other dianhydride monomers can also be used to cap terminal amino groups. The PABB_R_ precursor were capped with 6FDA and EG to obtain the PIBB_t-F_ and PIBB_t-F/E_ films by using the same process as described in the experimental section. The UV–vis spectra and optical performances are shown in Figure 3A and Table 2. The **λ_cut_** of the PIBB_t-F_ and PIBB_t-F/E_ films are both shorter than the PIBB_t-B_ and PIBBt-B_/E_ films_._ The **λ_cut_** blue-shift of the PIBB_t-F/E_ and PIBB_t-F_ films are higher than the PIBB_t-B/E_ and PIBB_t-B_ films_._ These results reveal that the electron cloud density of the terminal diamine segments is reduced more by 6FDA than the BPDA. It can be concluded that the 6FDA with electron-withdrawing groups can decrease the Morkit side reaction more efficiently. The steric hindrance groups in diamine monomer can inhibit electrophilic substitution as shown in Scheme 1, and 2,2′-bis (trifluoromethyl) benzidine (TFMB) and 6FDA were used as monomers to prepare PITF_R,_ PITF_t-F_, and PITF_t-F/E_ films using the same process as the PIBB_R,_ PIBB_t-F_, and PIBB_t-F/E_ films. UV–vis spectra of the PITF films are shown in Figure 3B, and their optical performances are listed in Table 2. The **λ_cut_** of the PITF_R,_ PITF_t-F_. and PITF_t-F/E_ films are subsequently blue-shifted, and the transparency sequentially increases. They show similar improvement patterns as the spectra of PIBB_R,_ PIBB_t-F_, and PIBB_t-F/E_ films. The difference is that all PITF_R,_ PITF_t-F_, and PITF_t-F/E_ films are colorless and transparent, meaning that the –CF_3_ group of TFMB can also sterically inhibit electrophilic substitution of 6FDA to TFMB during the synthesis process of PATF precursor and the heating imidization process of PITF film.

The electron withdrawing groups in diamine can also inhibit electrophilic substitution. Both 3,3′-Sulfonyldianiline (APS) and 6FDA were used as monomers to prepare PISF_R,_ PISF_t-F_, and PISF_t-F/E_ films. Their optical test results are shown in Figure 3C and Table 2. The –SO_2_– in APS was used as an electron-withdrawing group to decrease electron cloud density and electrophilic substitution reactivity of diamine monomers. The electrophilic substitution reactions of 6FDA to the APS segments becomes more difficult, thus, decreasing Morkit units during synthesis of PASF precursors and heating of PISF films. As expected, all PISF_R,_ PISF_t-F_, and PISF_t-F/E_ films are more colorless and transparent than PITFs and PIBBs.

As shown in Figure 3A–C, CPI films can be obtained from PITFs or PISFs. The **λ_cut_** of PIBB_t-F_, PITF_t-F_ and PISF_t-F_ are all shorter than PIBB_R_, PITF_R,_ and PISF_R_. This is mainly because the amino groups are capped by the blocker molecules 6FDA, while both colored –N=O and –NO_2_ chromophores are hardly produced during the heating process. The **λ_cut_** of the PIBB_t-F/E_ and PITF_t-F/E_ films are both shorter than that of PIBB_t-F_ and PITF_t-F_, respectively, revealing that few Morkit units are produced for the residual terminal anhydride groups capped with EG. While the **λ_cut_** of the PISF_t-F/E_ films are almost the same as that of PISF_t-F_, this result means that the electron-withdrawing groups of –SO_2_–can completely prevent the diamine segments from electrophilic substitution by anhydride groups. The EG blocker only improves the transmittance by reducing Rayleigh scattering on a nano-scale. In Figure 3D, the **λ_cut_** of PITF and PISF films are both shorter than PIBB films, the reason being that both the steric hindrance effect of –CF_3_ in TFMB and the electron-withdrawing groups of –SO_2_– in APS segments prohibit the electrophilic substitution of anhydride groups. Morkit unit formation can be decreased during synthesis of PATF and PASF precursors, as well as during heating of the PITF and PISF films. This is because the electron-withdrawing group of –SO_2_–/–CF_3_ decrease electron density and electrophilic substitution of the benzene ring in APS/TFMB segments of PASF/PATF. The Morkit units become difficult to produce. The results verify that the electron-withdrawing effect of –SO_2_– in APS is higher than the steric hindrance of –CF_3_ in TFMB, as shown in Table 2 and Figure 3D. Rayleigh scattering due to ordered structures at the nanometer level is also decreased by the blockers of 6FDA and EG, hence, the transparency of PIBB_t-F/E_, PITF_t-F/E_, PISF_t-F/E_, PIBB_t-F_, PITF_t-F_, and PISF_t-F_ films are all increased to higher than PIBB_R_, PITF_R,_ and PISF_R_ in the UV-vis region. An essential requirement for substrate materials used in organic light-emitting diodes devices is that light transmission should be higher than 80% at 430 nm; all capped PIs meet this condition.

#### 3.2.2. Thermal Properties of Polyamide Acid and Polyimide Films

**Figure 4:**
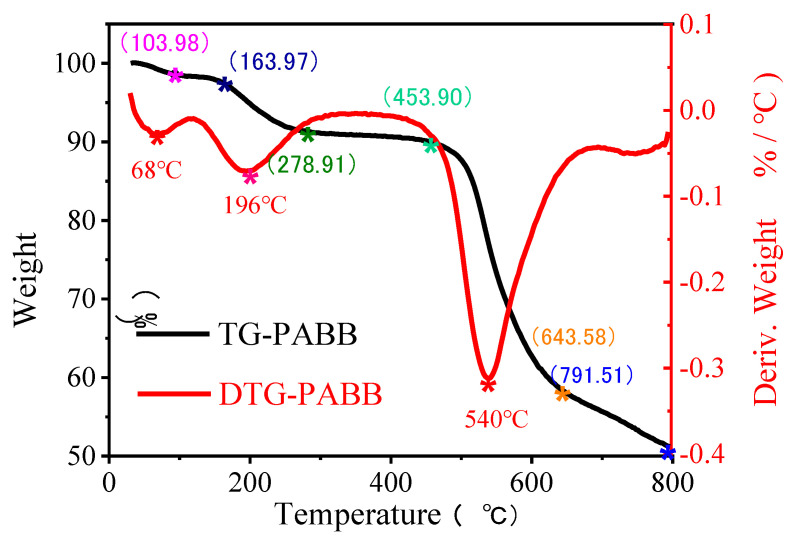
TGA thermograms of PABB resin.

In order to study the thermo-chemical reactions of the polyamide acid resin (PABB_R_) during the preparatory heating process of PIBB films, the polyamide acid solution (PABB_R_) was precipitated in deionized water, washed 3–5 times, and dried in a vacuum. The samples were evaluated by TGA (Figure 4), and the results are summarized in Table 3. There are three quick weight-loss stages in TGA and DTG curves for PABB resin. They correspond to the solvent H_2_O and DMAc in PABB volatilized at 25–103 °C (DTG 68 °C), the condensed H_2_O lost during the imidization process at 163–278 °C (DTG 196 °C), and the imide rings of PIBB thermally decomposed at 453–643 °C (DTG 540 °C). The C–N bond in the main chain of polyimide and the C–C bond in the amide ring are pyrolyzed and broken, free radicals and CO gas are generated, and then chain transfer occurs and, thus, mass begins to rapidly decline. There are also three slow weight-loss stages. The first slow weight-loss is recorded in the temperature range of 103–163 °C, likely representing the side reaction of generating Morkit units by the electrophilic substitution of terminal anhydride groups to diamine monomer chain segments as described in Scheme 2. The second slow weight-loss interval is 278–453 °C, which is a relatively stable pyrolysis and the removal process of terminal anhydride groups, carboxyl groups, or amino groups. The third slow weight-loss interval is 643–791 °C, which is the carbonization process of pyrolytic dehydrogenation of the benzene ring in the main polyimide chain. The residual carbon content at 780 °C is above 52%, which indicates that PIBB films have admirable thermal stability.

**Figure 5:**
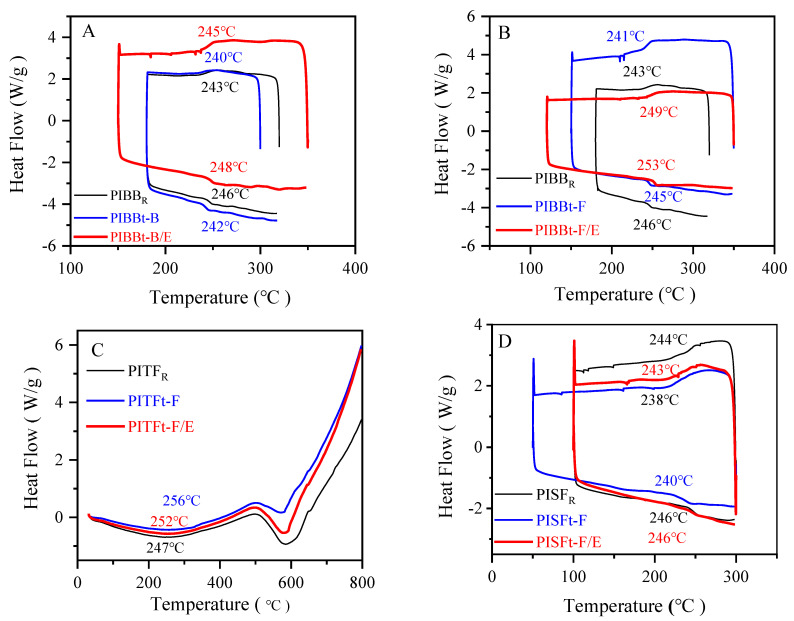
DSC thermograms of CPI films capped with different blockers: (**A**) PIBB_R_, PIBB_t-B_ and PIBB_t-B/E_; (**B**) PIBB_R_, PIBB_t-F_ and PIBB_t-F/E_; (**C**) PITF_R_, PITF_t-F_, and PITF_t-F/E_; (**D**) PISF_R_, PISF_t-F_ and PISF_t-F/E_.

**Figure 6:**
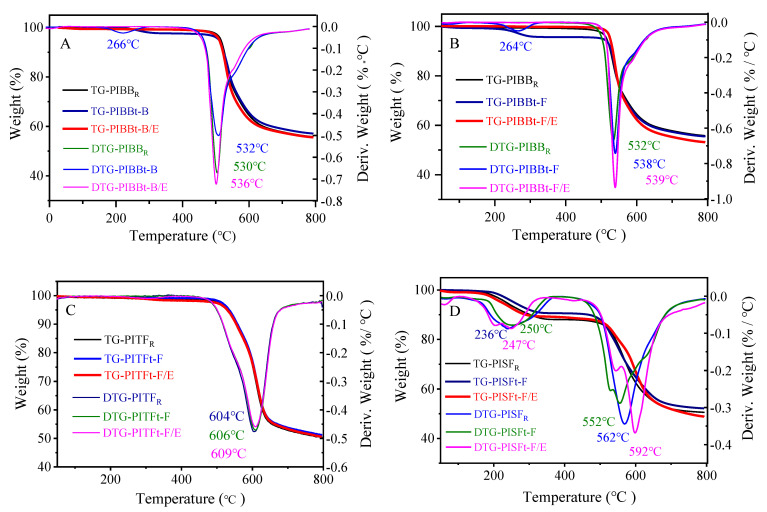
TGA thermograms of CPI films capped with different blockers: (**A**) PIBB_R_/PIBB_t-B_/PIBB_t-B/E_; (**B**) PIBB_R_/PIBB_t-F_/PIBB_t-F/E_; (**C**) PITF_R_/PITF_t-F_/PITF_t-F/E_; and (**D**) PISF_R_/PISF_t-F_/PISF_t-F/E_.

**Figure 7:**
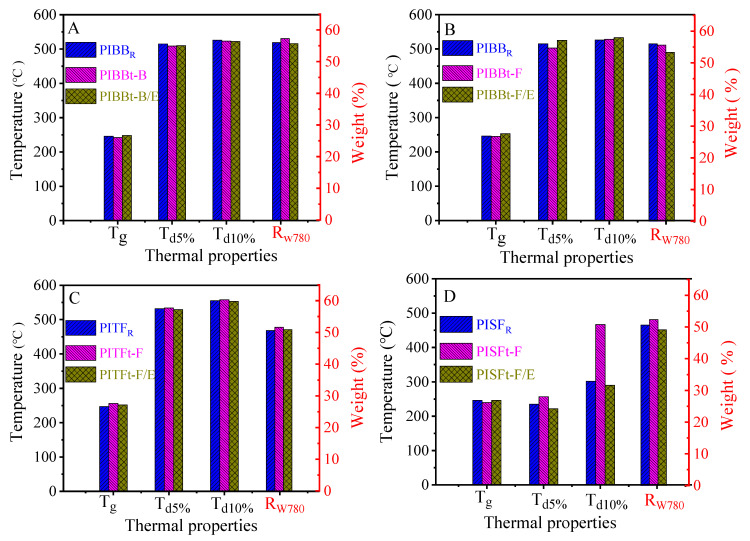
T_g_, T_d5%_, T_d10%,_ and R_w780_ comparison of the PI films capped with different blockers: (**A**) PIBB_R_/PIBB_t-B_/PIBB_t-B/E_; (**B**) PIBB_R_/PIBB_t-F_/PIBB_t-F/E_; (**C**) PITF_R_/PITF_t-F_/PITF_t-F/E_; and (**D**) PISF_R_/PISF_t-F_/PISF_t-F/E_.

Thermal properties of capped polyimide films (PIs) were evaluated by their glass transition temperatures (T_g_), determined by differential scanning calorimetry (DSC), shown in Figure 5, and by their decomposition temperatures with a 5% and 10% weight loss (T_d5%_, T_d10%_) observed by thermogravimetric analysis (TGA), as shown in Figure 6. The thermal properties of the PIBBs, PITFs, and PISFs are summarized in Figure 7 and Table 4.

The DSC thermograms of the PIBBs are presented in Figure 5A,B, where no apparent glass transitions (T_g_) are detected for all PIBBs below 350 °C. The T_g_ values measured at the second heating process are slightly higher than the first cooling process. This may be associated with further condensation or cross-linking between the tail groups of polyimides after the first heating process, while the temperature is still lower than the decomposition temperature. The T_g_ values measured for PIBBs are 242–253 °C. Since BPDA is a rigid structure and the polyimide ring is difficult to rotate, the polyimide film has good heat resistance. The T_g_ values of PIBB_t-B/E_ and PIBB_t-F/E_ films are a little higher than PIBB_R,_ because the PIBB molecular weight increases due to the coupling effect of EG (Figure 7A,B). The DSC curves of PITFs and PISFs are shown in Figure 5C,D and their thermal stability is similar to PIBBs’. The T_g_ values of PITF_t-B/E_ and PISF_t-F/E_ films are higher than PITF_R_ and PISF_R_ as the molecular weights are increased by the coupling effect of EG (Figure 7C,D). In general, T_g_ values of polyimide film are not impacted much by the two-step capping method, meaning that the end-capping PI films can be processed in a similar way to general PIs.

The TGA and DTG curves of PIBBs, PITFs, and PISFs can be seen in Figure 6A–D. The 5% weight-loss temperature (T_d5%_), 10% weight-loss temperature (T_d10%_), and residual carbon contents at 780 °C (R_w780_) are shown in Figure 7A–D. All PIBBs show good thermal stability with T_d5%_ at 503–525 °C, T_d10%_ at 522–533 °C, and even at 780 °C, thermogravimetry is above 53%. PIBB_t-B_ capped with BPDA shows a 2.2% lower weight-loss than PIBB_R_ and PIBB_t-B/E_, while the PIBB_t-F_ capped with 6FDA shows 4.0% less weight-loss than PIBB_R_ and PIBB_t-F/E_ (Figure 7A,B). The differences in thermal stability are due to small molecule evaporation or anhydride group decomposition at 200–300 °C. On the other hand, the blocker EG increases PAA molecular weight by coupling. The thermal stability of PIBB_t-B/E_ and PIBB_t-F/E_ are both higher than PIBB_t-B_ and PIBB_t-F_ under the pyrolysis temperature of PIBBs. The decomposition of PIBB_t-B_ and PIBB_t-F_ chains generally appear at lower temperatures than that of PIBB_t-B/E_ and PIBB_t-F/E_, due to the unstable anhydride groups.

High fluorine content reduces the decomposition rate of PIBB_t-F_. The fully aromatic PIBB_t-F/E_ with –CF_3_ groups shows better thermal stability with higher Td_5%_ at ∼525 °C than PIBB_t-B/E_ at ∼510 °C. The TGA curves of PITFs are shown in Figure 6C and the thermal stability is similar to PIBBs (Figure 7C), yet the TGA curves of PISFs in Figure 6D reveal that PIBBs are less stable than PITFs, due to the presence of–SO_2_– in APS, as shown Figure 7D.

#### 3.2.3. Mechanical Properties of PI Films

**Figure 8:**
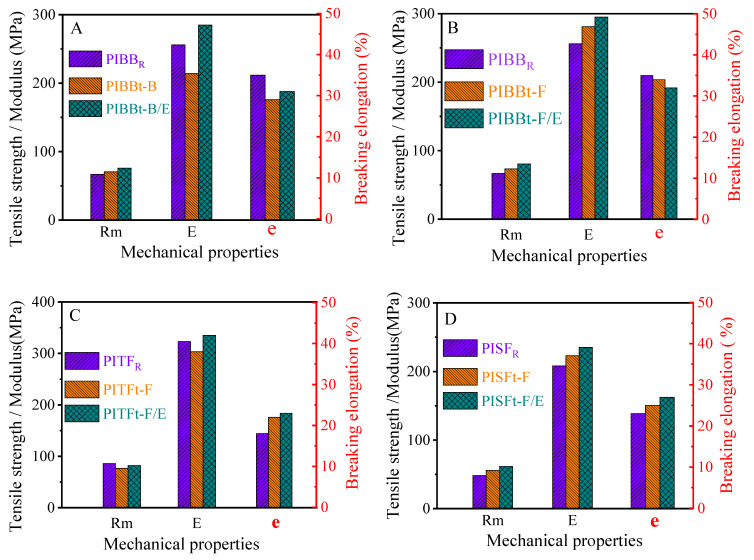
Tensile strength (Rm/MPa), tensile modulus (E/MPa), and elongation at break (e/%) comparison of the PI films capped with different blockers: (**A**) PIBB_R_/PIBB_t-B_/PIBB_t-B/E_; (**B**) PIBB_R_/PIBB_t-F_/PIBB_t-F/E_; (**C**) PITF_R_/PITF_t-F_/PITF_t-F/E_ and (**D**) PISF_R_/PISF_t-F_/PISF_t-F/E_.

As shown in Figure 8 and Table 5, the tensile strength and modulus of PIBB_R_ are increased by the coupling effect of dianhydride and EG blockers. The fluorine-containing PIBB_t-F/E_ exhibit enhanced mechanical properties compared to PIBB_t-B/E_, with tensile strength and modulus of 80.7 MPa and 295 Mpa, respectively. The improvement is likely due to the better mechanical properties of 6FDA blockers and the coupling effect to PIBBs backbones. The mechanical properties of the PITFs and PISFs are similar to PIBBs. Apparently, the mechanical properties of the capped PI films are enhanced by the two-step capping method.

#### 3.2.4. Surface Energy of CPI Films

**Figure 9:**
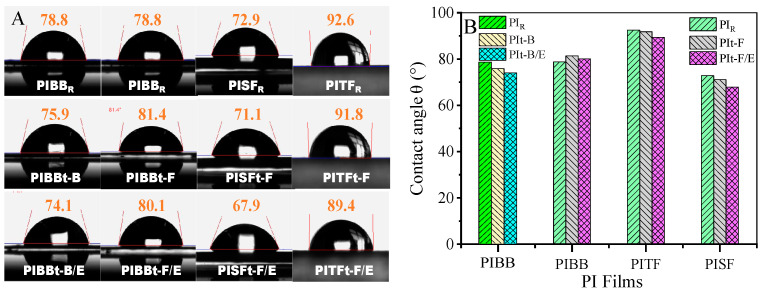
Contact angle images of water droplets on films (**A**) and contact angle comparison of the PIBBs, PITF_S,_ and PISFs films (**B**).

Contact angle measurements of the polymer are generally conducted to investigate surface hydrophobicity. The test results are shown in Figure 9 and Table 6. A decrease in contact angle means surface hydrophilicity is increased. It can be observed that the polar blockers of dianhydride make PIBB_t-B_, PITF_t-F_, and PISF_t-F_ more hydrophilic than the PIBB_R,_ PITF_R,_ and PISF_R_ films. It can also be noted that the polar blockers of dianhydride and EG make the PIBB_t-B/E_, PIBB_t-F/E_, PITF_t-F/E_, and PISF_t-F/E_ films more hydrophilic than the PIBB_t-B_, PITF_t-F_, and PISF_t-F_ films. PIBB_t-F/E_ is more hydrophobic than PIBB_R_ due to the –CF_3_ groups, which also remarkably improve the hydrophobicity of PITFs.

## 4. Conclusions

In summary, it is found that Morkit units, N-Oxide, and Azo chromophores are all responsible for yellowing of PI, except for the CTC effect. The terminal amino groups of PAA are capped by the dianhydride monomers, hence, yellow azo– compounds and nitro-compounds are oxidized from diamine segments with tail amino groups are decreased. Furthermore, the electron cloud density of terminal diamine segments are also decreased by the amido bond with blockers of the dianhydride monomers. This means the electrophilic substitution of the tail anhydride groups to terminal diamine segments becomes difficult, thus, making it difficult for the Morkit units to form. The cut-off wavelength of the capped PIBB, PITF, and PISF films then become shorter and the transmittance is increased. Moreover, the strong electron-withdrawing group can decrease the electron density of neighboring atoms and inhibit Morkit unit formation more effectively. Subsequently, terminal anhydride groups are capped by EG and electrophilic substitution reactions to diamine segments of terminal anhydride groups decrease, ultimately leading to the decreased formation of Morkit units. Rayleigh scattering due to the ordered and rigid nanoscale structures of PI is also lowered by the flexible EG blockers and different dianhydride monomers, thus, improving the transparency of CPI in the UV-vis region. The capped PIBB, PITF, and PISF molecular weights and mechanical tensile properties can be increased by the coupling effects of blockers. The final-stage, capped PIBB, PITF, and PISF films exhibit high thermal stability without apparent changes in their DSC curves below decomposition temperatures. This research shows that the electron-withdrawing effect, steric hindrance effect, and the capping of residual groups can be useful strategies to decrease the Morkit units and chromophores when preparing CPIs.

## Figures and Tables

**Table 1:** Optical performance of PIBB films after different temperature thermal treatment.

Films	Temp. (°C)	70.0	100	150	200	250
PIBB_R_	λ_cut_ (nm)	362	370	398	393	414
PIBBt-B	λ_cut_ (nm)	373	395	400	402	402
PIBBt-B/E	λ_cut_ (nm)	371	386	401	405	400
PIBB_R_	T_450_ (%)	92.6	81.2	48.0	55.9	29.9
PIBBt-B	T_450_ (%)	89.6	63.4	55.2	42.3	52.0
PIBBt-B/E	T_450_ (%)	89.6	72.4	57.4	43.3	55.4
PIBB_R_	T_max_ (%)	99.2	99.4	97.6	96.4	93.7
PIBBt-B	T_max_ (%)	96.5	96.4	96.2	93.9	97.8
PIBBt-B/E	T_max_ (%)	96.6	96.7	93.8	94.7	98.9

**Table 2:** Optical properties of PIBBs, PITFs, and PISFs films.

PIs	Diamine/Dianhydride	Blockers	η_inh_	λ_cut_ (nm)	T_450_ (%)	T_max_ (%)
PIBB_R_	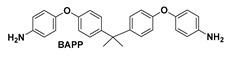	None	0.5613	414	29.9	93.7
PIBB_t-F_		6FDA	0.4875	388	71.3	97.0
PIBB_t-F/E_	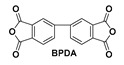	6FDA/EG	0.3425	370	77.0	97.5
PITF_R_	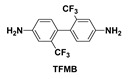	None	0.1599	345	90.2	94.7
PITF_t-F_		6FDA	0.1561	340	93.6	96.3
PITF_t-F/E_	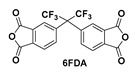	6FDA/EG	0.2423	335	97.1	98.2
PISF_R_	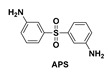	None	0.1643	341	79.6	84.2
PISF_t-F_		6FDA	0.1431	336	83.8	87.1
PISF_t-F/E_	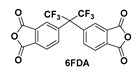	6FDA/EG	0.2437	334	91.6	94.8

**Table 3:** TGA results of PABB resin.

Stage	No. 1	No. 2	No. 3	No. 4	No. 5	No. 6
Temp. (°C)	25–103	103–163	163–278	278–453	453–643	653–791
Weight loss %	100–98	98–97	97–91	91–90	90–58	58–51
Lost groups	H_2_O	H_2_O	H_2_O	CO_2_	−CONCO−	H_2_
Number	Many	Few	Many	Few	Many	Many
Speed	Quick	Slowly	Quick	Slowly	Quick	Slowly
Thermo- reaction	Solvent evaporation	Morkit	Imidization	Anhydride pyrolysis	Imide ring pyrolysis	Carbonation

**Table 4:** Thermal properties of PIBBs, PITFs, and PISFs.

Parameters	PIBB R	PIBB t-B	PIBB t-B/E	PIBB t-F	PIBB t-F/E	PITF R	PITF t-F	PITF t-F/E	PISF R	PISF t-F	PISF t-F/E
T_g_	246	242	248	245	253	247	256	252	246	240	246
T_d5%_	515	509	510	503	525	532	534	529	235	257	222
T_d10%_	526	523	522	528	533	555	558	553	302	467	290
R_w780_	56.0	57.3	55.7	55.6	53.3	50.6	51.6	50.9	50.6	52.3	49.1

**Table 5:** Mechanical properties of PIBBs, PITFs, and PISFs.

Parameters	PIBB R	PIBB t-B	PIBB t-B/E	PIBB t-F	PIBB t-F/E	PITF R	PITF t-F	PITF t-F/E	PISF R	PISF t-F	PISF t-F/E
Rm (MPa)	66.9	70.5	75.8	73.5	80.7	86.0	76.8	82.0	48.3	55.6	61.5
E (MPa)	256	214	285	281	295	323	303	335	208	223	235
e (%)	35	29	31	34	32	18	22	23	23	25	27

**Table 6:** Contact angles of PIBBs, PITFs, and PISFs.

Contact Angle	PIBB R	PIBB t-B	PIBB t-B/E	PIBB t-F	PIBB t-F/E	PITF R	PITF t-F	PITF t-F/E	PISF R	PISF t-F	PISF t-F/E
( ͦ )	78.8	75.9	74.1	81.4	80.1	92.5	91.8	89.4	72.9	71.1	67.9

## Data Availability

The data presented in this study are available on request from the corresponding author.
